# Physiological Responses of Pacific White Shrimp *Litopenaeus vannamei* to Temperature Fluctuation in Low-Salinity Water

**DOI:** 10.3389/fphys.2019.01025

**Published:** 2019-08-13

**Authors:** Zhenlu Wang, Yuexin Qu, Muting Yan, Junyi Li, Jixing Zou, Lanfen Fan

**Affiliations:** ^1^Department of Aquaculture, College of Marine Sciences, South China Agricultural University, Guangzhou, China; ^2^Joint Laboratory of Guangdong Province and Hong Kong Region on Marine Bioresource Conservation and Exploitation, South China Agricultural University, Guangzhou, China

**Keywords:** *Litopenaeus vannamei*, temperature, endoplasmic reticulum stress, apoptosis, self-regulation

## Abstract

Temperature is a significant environmental factor in aquaculture. To investigate the physiological responses during temperature fluctuation (28~13°C), experimental shrimps (*Litopenaeus vannamei*) were treated with gradual cooling from acclimation temperature (AT, 28°C) to 13°C with a cooling rate of 7.5°C/day and rose back to 28°C at the same rate after 13°C for 24 h. Hepatopancreas histological changes, plasma metabolites concentrations, relative mRNA expression of unfolded protein response (UPR) pathway and apoptosis in hepatopancreas and hemocyte were investigated. The results showed that with the decline of temperature, the number and volume of the secretory cells in hepatopancreas increased significantly, the tubule lumen appeared dilatated, and the epithelial cell layer became thinner. The contents of glucose (Glu) significantly decreased to the minimum value of 13°C for 24 h. The contents of triglyceride (TG), total cholesterol (TC), and total protein (TP) increased and reached the peak of 13°C for 24 h. Alkaline phosphatase (ALP) and alanine aminotransferase (ALT) activities in plasma reached the lowest and highest value in 13°C, respectively. The expressions of all genes related to UPR and apoptosis in the hepatopancreas and hemocytes were significantly changed during the cooling process and reached the highest level of 13 and 13°C for 24 h, respectively. During re-warming stage, the histopathological symptoms got remission and each of the plasma metabolite concentrations and gene expressions returned to AT levels. These results revealed that pacific white shrimp can adapt to a certain level of temperature fluctuation by self-regulation.

## Introduction

The pacific white shrimp *Litopenaeus vannamei*, with a wide range of salt-tolerance, rapid growth, and other characteristics suitable for intensive aquaculture, has become one of the most important aquaculture shrimps in the world. However, a variety of environmental stimuli affect the growth of shrimp, such as changes in pH (Han et al., 2018a), salinity (Li et al., 2008; Wang et al., 2016), dissolved oxygen (DO) (Han et al., 2018b), temperature (Madeira et al., 2015), and pollutants like nitrite, ammonia, and sulfide (Duan et al., 2018b).

Since the start of global climate change, various extreme climates have happened frequently. Previous studies have indicated that many extreme weather events which were associated with drastic temperature fluctuation can directly affect the growth, physiological performance, and survival of animals (He et al., 2018; Zhang et al., 2019). It has been studied that temperature changes may lead to growth arrest, stoppage of feeding, and swimming or even death in 13°C (Fan et al., 2013; Huang et al., 2017; Xu et al., 2018). Our previous study has indicated that glucose-regulated protein 78 kDa (GRP78) was significantly up-regulated in the hepatopancreas of *L. vannamei* under 13°C for 24 h cold-stress (Fan et al., 2016). GRP78, also known as immunoglobulin-binding protein (BIP), is a central regulator of endoplasmic reticulum stress (ERS) and regulated the process of unfolded protein response (UPR) and apoptosis (Dejean et al., 2006; Nakka et al., 2010). At present, studies on ERS are mainly focused on mammals and the UPR pathway (Cao and Kaufman, 2012).

UPR is a self-protective mechanism which can promote cell survival in response to ERS. It includes three classical signaling pathways: the activating transcription factor 6 (ATF6) pathway, the inositol-requiring enzyme-1-X-box binding protein 1 (IRE1-XBP1) pathway, and the protein kinase RNA (PKR)-like ER kinase-eIF2α (PERK-eIF2α) pathway (Mori, 2009; Costa et al., 2011). In addition, apoptosis signals will be generated if stress is prolonged for protecting the organism by eliminating the damaged cells. Apoptosis signal-regulated kinase 1 (ASK1) is essential for the continuous activation of c-Jun NH2-terminal kinases (JNK) and induces cell apoptosis (Tobiume et al., 2001). Cysteine-containing aspartate-specific proteases (caspases) are a family of proteases that perform apoptosis in animals. Apoptosis mediated by ERS triggers a specific cascade of caspase 12, 9, and 3, and the activation of caspase 3 (CASP3) indicates that apoptosis has entered an irreversible stage (Morishima et al., 2002). In invertebrates, UPR is widely recognized as the key to ER stress response (Chen and He, 2019). However, studies of the UPR signaling pathway and apoptosis in *L. vannamei* mainly focused on the immune function, especially in response to WSSV infection (Chen et al., 2012; Wang et al., 2013; Xu et al., 2014; Yuan et al., 2016, 2017, 2018). UPR in response to temperature fluctuation has not been reported.

Additionally, it has been identified that hepatopancreas histology could be used to monitor the impact of a stressed environment, showing ultrastructural alterations at the early stage of stress (Collins, 2010). Environmental changes like pH stress can cause change or damaged of hepatopancreas cells (Tao et al., 2016). However, it is still not clear about the change of hepatopancreas histology during temperature fluctuation process.

In the present study, based on the statistics of weather conditions from the winter (November, December and January) in Guangdong from 2017 to 2018 (China Meteorological Administration, www.cma.gov.cn), we found that the average daily temperature difference of winter in Guangdong was 7.52°C. The annual cold wave causes huge economic losses to the *L. vannamei* breeding industry in China. However, little was known about the responses of the shrimp during the process of temperature gradual cooling and warming. Thus, we investigated (1) histological section of the hepatopancreas, (2) metabolite concentrations of plasma, and (3) UPR gene and cell apoptosis gene expressions of hepatopancreas and hemocyte in *L. vannamei* during temperature fluctuations. These results could provide valuable reference to analyze the adaptation mechanism of the shrimp in response to temperature fluctuation.

## Materials and Methods

### Experimental Shrimp and Culture Conditions

The experimental shrimps, with an average weight of 5.4 ± 0.7 g, were obtained from a commercial farm in Panyu (Guangdong, China). The shrimps were immediately transported to the lab and acclimated in 500 L filtered, aerated (oxygen pump, HAP-120, HAILEA, Guangdong, China) seawater tanks (Guanzhong, K500 L, Jiangsu, China) at least 4 days before experiments. During the acclimation stage, the water salinity and temperature in tanks were consistent with that of the culture ponds (salinity 5‰, pH 8.3 ± 0.1 and temperature 28 ± 1°C). Commercial shrimp feeds (Haida Group Feed, Jieyang, China) were given two times per day (5% of shrimp body weight per time).

### Treatment

Sixty-three healthy shrimps were randomly divided into three replicate tanks. They were placed in an artificial climate incubator (Laifu, Ningbo, China), and the water temperature was decreased from acclimation temperature (AT, 28°C) to 13°C with a cooling rate of 7.5°C/day (2.5°C/8 h). After 13°C for 24 h, the water temperature rose back to 28°C at the same rate.

### Sample Collection

#### Tissue Slice

At each temperature point [28, 23, 18, 13, and 13°C for 24 h during cooling process, 18 and 28°C during return process (r18 and r28°C)], the whole hepatopancreas of one shrimp from each tank were dissected from the cephalothoraxes and fixed with 4% paraformaldehyde (Biosharp, China) for tissue fixation and then stored in 4°C for paraffin sections by Servicebio (Wuhan, China).

#### Plasma and Gene Expression Analysis

Hemolymph was extracted from the ventral sinus of shrimp at each temperature point as same as tissue slice, using a 1 ml sterile syringe containing an equal volume of ice-cold anticoagulant solution (27 mM trisodium citrate, 385 mM sodium chloride, 115 mM glucose, pH 7.5). Hemolymph of two shrimps from each tank was mixed as one sample, three repeats. After being centrifuged at 3000 rpm (844*g*) for 10 min in 4°C, the supernatant fluid was immediately stored in −80°C for analysis of plasma metabolite concentrations. The pelleted hemocytes were collected, instantly frozen in liquid nitrogen and then stored at −80°C for analysis of gene expression (Xu et al., 2018). After hemolymph sampling, hepatopancreases were dissected, frozen in liquid nitrogen, and stored in −80°C for gene expression analysis.

### RNA Extraction and cDNA Synthesis

Total RNA was extracted from hemocytes and hepatopancreases using RNAiso Plus reagent (TaKaRa, Japan) following the manufacturer’s protocol. RNA quality was assessed by electrophoresis on 1.0% agarose gel, and concentration was tested by mySPEC (VWR, USA). Total RNA was purified, and first-strand cDNA was synthesized using ReverTra Ace® qPCR RT Master Mix with gDNA Remover (TOYOBO, Shanghai) according to the manufacturer’s instructions.

### Real-Time Quantitative PCR

The SYBR Green real-time Polymerase Chain Reaction (PCR) assays were carried out on a CFX Connect™ Real-Time System (Bio-Rad) using THUNDERBIRD® SYBR® qPCR Mix (TOYOBO). Previous studies showed that the expressions of β-actin were constant after environmental stimuli such as ammonia (Duan et al., 2018a), dissolved oxygen (Han et al., 2018b), and pH stress (Han et al., 2018a). Therefore, we used β-actin as the housekeeping gene, and specific primer sequences were designed based on the coding sequence of the target genes using Primer Premier 6.0 software (Table 1). The real-time PCR program was 95°C for 1 min, followed by 40 cycles of 95°C for 15 s, 60°C for 15 s, and 72°C for 45 s, followed by 1 step of 95°C for 10 s. Melting curves were obtained by increasing the temperature from 65 to 95°C (0.5°C/s) to denature the double-stranded DNA. The relative mRNA expressions were calculated by the comparative Ct method (2^−ΔΔCt^).

**Table 1:** The real-time PCR primers used in this study.

Primer names	Nucleotide sequences (5′–3′)	Protein ID
LvGRP78-F	TCATTGCCAACGACCAGGGT	AFQ62791.1
LvGRP78-R	TCCGATGAGACGCTTGGCAT	
LvPERK-F	TCCTGACATCATCATTATCATCTCC	XP_027239142.1
LvPERK-R	TGAAGCTCATGCTCTCTGCCAATCC	
LveIF2α-F	GGAACCTGTCGTTGTCATCAGAGTAG	AGI97278.1
LveIF2α-R	AGAAGCTCTCCAACATGCCGAATG	
LvATF4-F	GCCACGATTCAAGATGCTGC	AGI97279.1
LvATF4-R	TCCTCCTCGTCCATGCCATA	
LvATF6-F	CTGTTGGGACAAGGACCATAAGC	AYM00394.1
LvATF6-R	GAATTGTAGGTGTGGCAGCTGTTA	
LvIRE1-F	TGGTGAGAAGCAGCTTGTGTTGG	AFQ62792.1
LvIRE1-R	ACTGTTGATGAAGAGCCACTTGTAGC	
LvXBP1-F	GTGGATCAGCAGTATCCCAACC	AFQ62793.1
LvXBP1-R	TGCCAAGGCAGCTGTATTGA	
LvCasp3-F	ACATTTCTGGGCGGAACACC	AGL61582.1
LvCasp3-R	GTGACACCCGTGCTTGTACA	
LvASK1-F	GCTGTGTTGAAGTCCGAGGAGAAG	AKI88007.1
LvASK1-R	AGCCAAGCAACCAACTCCATATCG	
LvActin-F	GACTACCTGATGAAGATCC	AAG16253.1
LvActin-R	TCGTTGCCGATGGTGATCA	

### Statistical Analyses

All the data were presented as mean ± SD of triplicates. Data were statistically analyzed by SPSS 19.0 with one-way ANOVA and Tukey test. *p* < 0.05 was significant difference.

## Results

### Hepatopancreas Histological Analysis

**Figure 1:**
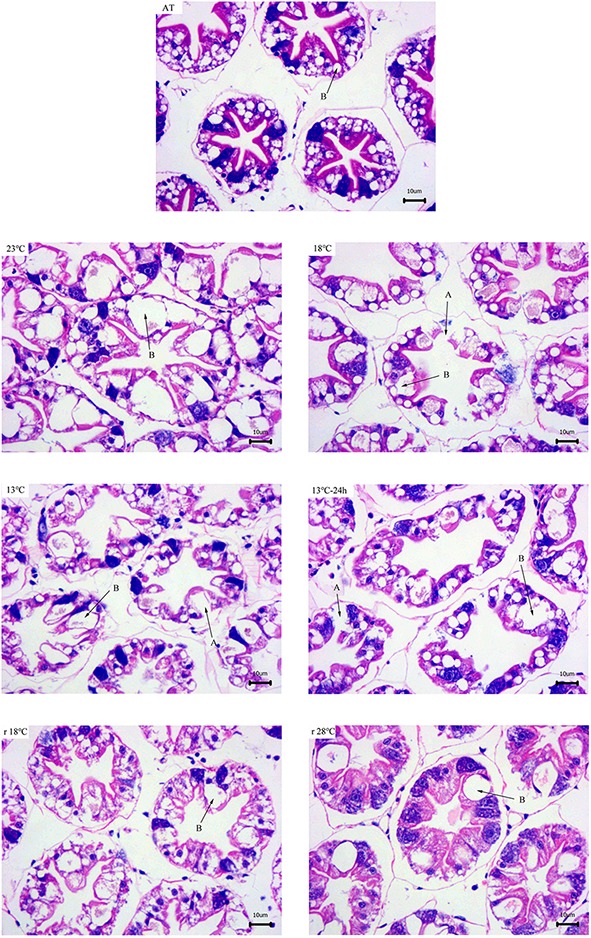
Hepatopancreas tissue structure (×400) of *L. vannamei* with HE dying during temperature fluctuation. AT represents acclimation temperature (28°C), and r18 and r28°C represent 18 and 28°C in temperature return process, respectively. The letters in the figure indicate: A, ruptured cells; B, B-cell.

According to the results of hepatopancreas with HE staining, the hepatocytes in 28°C exhibited the well-organized tubules. With the decrease of temperature, stellate tubule lumen appeared dilatation, and some vacuoles appeared and ruptured to make the epithelial cell layer thinner. The secretory cells (“blasenzellen”, B-cells), which are the main site for synthesis of digestive enzymes, typically contain a single large secretory vesicle. The number and volume of B-cells significantly increased during the cooling process. All these symptoms got remission during the temperature return process (Figure 1).

### Plasma Analysis

**Figure 2:**
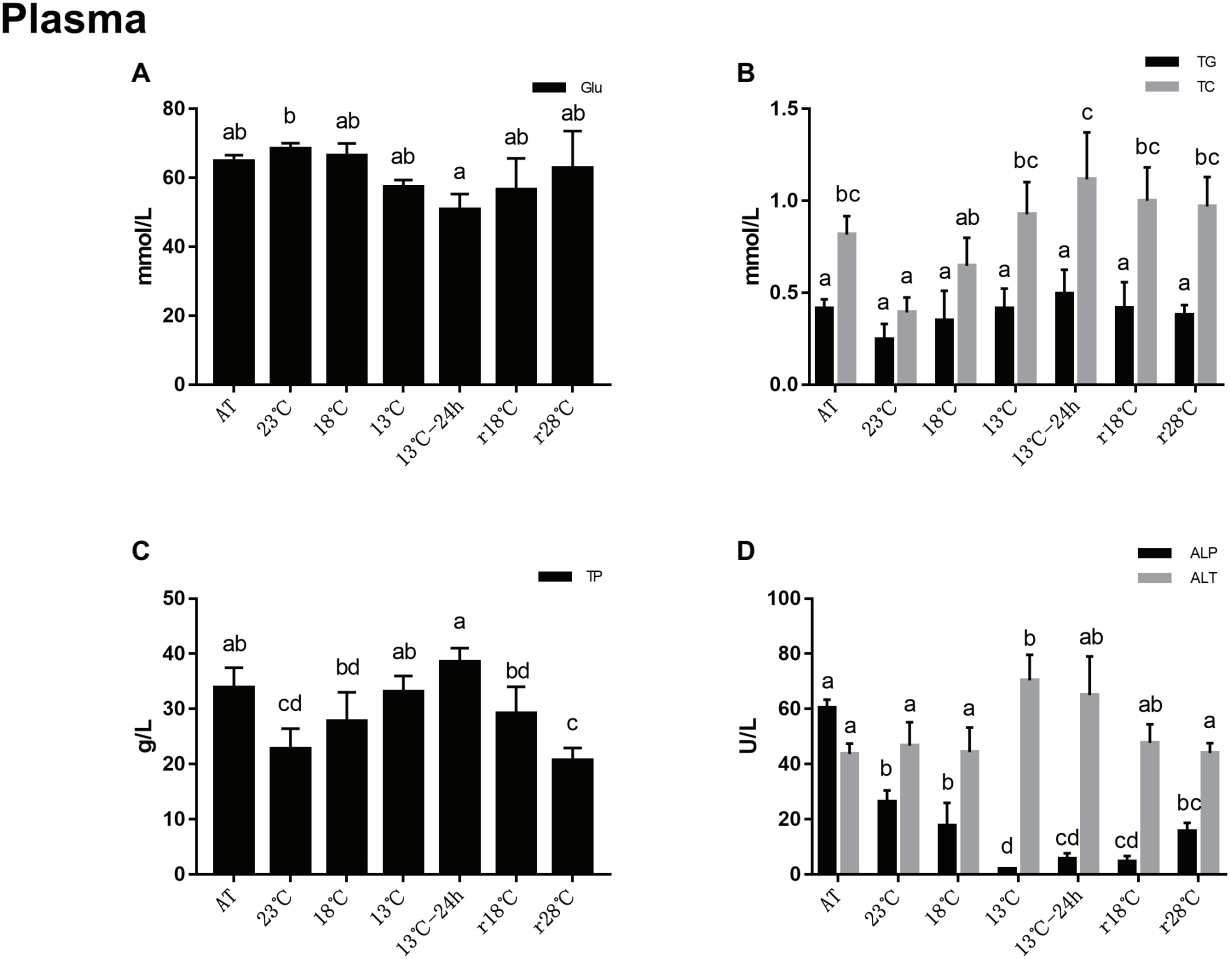
Contents of glucose (Glu, **A**), triglyceride (TG) and total cholesterol (TC) **(B)**, total protein (TP, **C**) and alkaline phosphatase (ALP) and alanine aminotransferase (ALT) **(D)** activities in plasma of *L. vannamei* during temperature fluctuation. AT represents acclimation temperature (28°C), r18 and r28°C represent 18 and 28°C in temperature return process, respectively. The bars represent the mean ± S.D. (*n* = 3). Statistical significance was calculated by one-way ANOVA. Bars with different letters indicate statistical differences (*p* < 0.05).

Analysis of plasma metabolite concentrations is shown in Figure 2. Compared to the AT group, the contents of glucose (Glu) decreased to the minimum value (50.78 mmol/L) at 13°C for 24 h. After temperature rose back to 28°C, it recovered to the level nearly of AT (r28°C = 62.82 mmol/L, AT = 64.81 mmol/L) (Figure 2A). The contents of triglyceride (TG) and total cholesterol (TC) decreased after cooling and reached the lowest (TG = 0.25 mmol/L, TC = 0.39 mmol/L) at 23°C, and then increased gradually and reached the peak of 0.49 mmol/L and 1.12 mmol/L, respectively, in 13°C for 24 h (Figure 2B), while the change of TG contents was not statistically different with the AT group. Changes of total protein (TP) content during the cooling process were similar to the TC and TG. During the return process, the content of TP reduced to 20.67 g/L in r28°C (Figure 2C). Alkaline phosphatase (ALP) activities in plasma decreased significantly after cooling and reached the lowest (2 U/L) at 13°C. After temperature rose back to 28°C, ALP activities increased to 15.67 U/L, which is near the activities at 18°C (17.67 U/L) in the cooling process. Alanine aminotransferase (ALT) activities remained stable from AT to 18°C and then significantly increased and reached the highest level (70.33 U/L) at 13°C. During the return process, ALT activities decreased to the level near AT (43.67 U/L) (Figure 2D).

### Unfolded Protein Response and Related Apoptosis Gene Expression to Temperature Fluctuation in the Hepatopancreas

**Figure 3:**
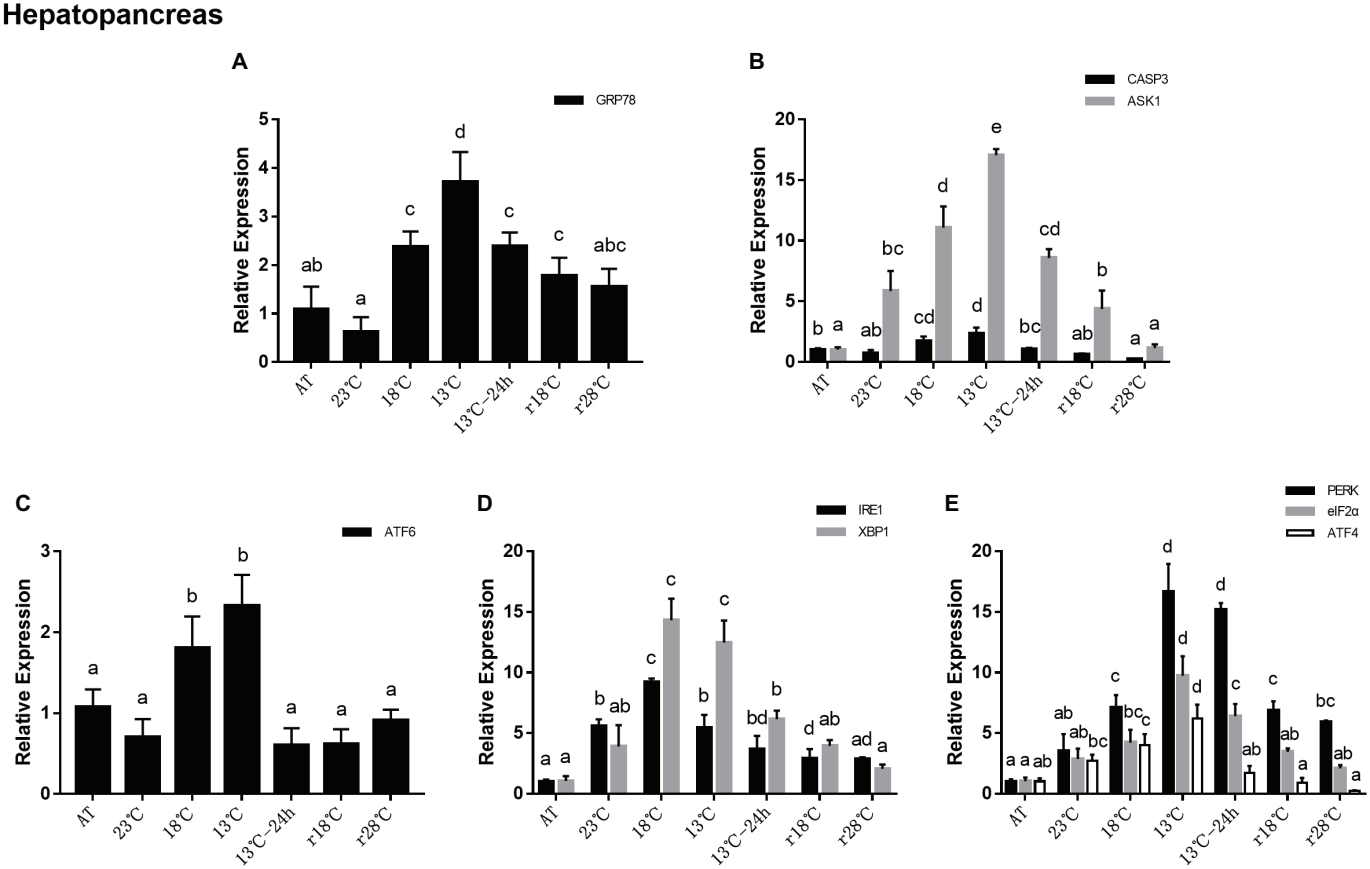
Relative expression of UPR and apoptosis related genes in hepatopancreas during temperature fluctuation. Water temperature was changed from 28 to 13°C with a cooling rate of 7.5°C/day. After 13°C for 24 h, the water temperature rose back to 28°C at the same rate. AT represents acclimation temperature (28°C), r18 and r28°C represent 18 and 28°C in temperature return process, respectively. The relative mRNA expression levels of GRP78 **(A)**, apoptosis related genes (CASP3, ASK1) **(B)**, ATF6 pathway (ATF6) **(C)**, IRE1 pathway (IRE1, XBP1) **(D)**, and PERK pathway (PERK, eIF2α, ATF4) **(E)** were compared with those at AT. The bars represent the mean ± S.D. (*n* = 3). Statistical significance was calculated by one-way ANOVA. Bars with different letters indicate statistical differences (*p* < 0.05).

In the hepatopancreas, the relative expressions of GRP78 increased significantly at 18°C and reached the peak at 13°C, which is about fourfold that at AT. During the next 24 h maintaining in 13°C and the return process, GRP78 expressions significantly decreased and they were near to the level of AT in r28°C (Figure 3A). Expressions of apoptosis related genes including CASP3 and ASK1 showed the same trend with the PERK sub-pathway and their highest expression levels appeared in 13°C (Figure 3B). In UPR, expressions of ATF6 showed the same trend found in GRP78 (Figure 3C). In the IRE1 sub-pathway, expressions of IRE1 and XBP1 reached the peak at 18°C (Figure 3D). In the PERK sub-pathway, expressions of PERK, eIF2α, and ATF4 increased gradually during the cooling process, and the highest expressions (16.67, 9.74, and 6.21 folds compared with that at AT, respectively) appeared at 13°C and then decreased significantly. There was no obvious difference among the expressions of eIF2α and ATF4 between r28°C and AT (Figure 3E).

### Unfolded Protein Response and Related Apoptosis Gene Expression to Temperature Fluctuation in Hemocyte

**Figure 4:**
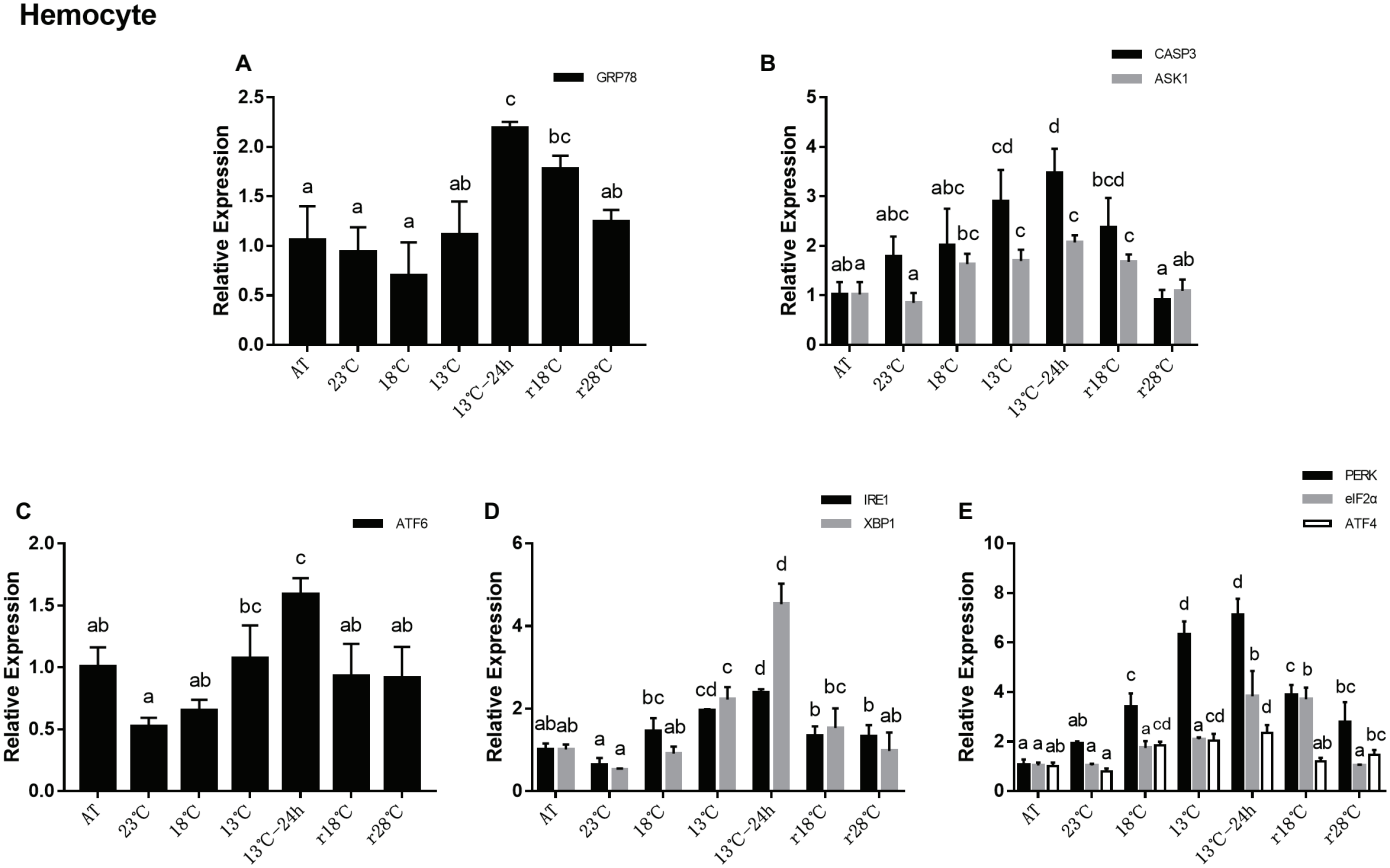
Relative expression of UPR- and apoptosis-related genes in hemocyte during temperature fluctuation. Water temperature was changed from 28 to 13°C with a cooling rate of 7.5°C/day. After 13°C for 24 h, the water temperature rose back to 28°C at the same rate. AT represents acclimation temperature (28°C), r18 and r28°C represent 18 and 28°C in temperature return process, respectively. The relative mRNA expression levels of GRP78 **(A)**, apoptosis-related genes (CASP3, ASK1) **(B)**, ATF6 pathway (ATF6) **(C)**, IRE1 pathway (IRE1, XBP1) **(D)**, and PERK pathway (PERK, eIF2α, ATF4) **(E)** were compared with those at AT. The bars represent the mean ± S.D. (*n* = 3). Statistical significance was calculated by one-way ANOVA. Bars with different letters indicate statistical differences (*p* < 0.05).

In the hemocyte, the expression level of GRP78 remained stable from AT to 13°C and then significantly increased in 13°C for 24 h, which is more than twofold the level in AT. After temperature rose back, expressions of GRP78 were approximate to the level of AT in r28°C (Figure 4A). For genes related to apoptosis, expressions of CASP3 and ASK1 showed the same trend with the PERK sub-pathway (Figure 4B). In UPR, the expressions of ATF6 decreased significantly after cooling in 23°C and then increased gradually and reached the highest level in 13°C for 24 h compared with the expression in AT. After temperature rose back, it returned to nearly the level of AT (Figure 4C). In the IRE1 sub-pathway, IRE1 and XBP1 showed a similar trend as ATF6 (Figure 4D). In the PERK sub-pathway, expressions of PERK, eIF2α, and ATF4 increased gradually after cooling, reaching at 13°C for 24 h approximately seven-, four-, and twofold, respectively, of the levels found in AT, but after temperature rose back the expressions decreased (Figure 4E).

## Discussion

Global climate change is impacting marine and estuarine aquaculture. It is generally known that coastal marine systems are some of the most important ecologically and socio-economically on the planet. Temperature, as one of the interacting climatic variables, will drive future ecological changes in marine systems. Understanding how temperature change will affect the aquatic organisms is a key issue worldwide. *L. vannamei*, mainly distributed in the sea of Ecuador and introduced to China in 1988, is one of the most important aquaculture shrimps in the world. As its origins are tropical, temperature fluctuation is a serious challenge to its survival, growth, and distribution (Peng et al., 2015; Cottin et al., 2016).

In this study, the hepatopancreas histological changes, plasma metabolites concentrations, and relative mRNA expression in the UPR signaling pathway and apoptosis genes induced by ERS in *L. vannamei* during cold stress were studied.

### Hepatopancreas Histological Change During Temperature Fluctuation

The hepatopancreas as a vital organ of crustaceans is involved in excretion, molting, diverse metabolic activities, and storage of energy reserves (Yepiz-Plascencia et al., 2000; Verri et al., 2001). In this study, the number and volume of B-cells in hepatopancreas tubules was significantly increased after suffering cold stress. This may be related to the fact that B-cells are the main site of absorption and digestion of nutrients (Almohanna and Nott, 1989; Wang et al., 2016). We suspected that the high rate of synthesis and release of digestive enzymes in B-cells accelerated the mobilization of nutrients in hepatopancreas tubules, by which shrimp can adapt to environmental stress.

The hepatopancreas of shrimp has high self-repairing ability. *L. vannamei* can repair hepatopancreas injury after long-term exposure to low zinc (Jui-Pin et al., 2008) and low pH (Han et al., 2018a). The hepatopancreas weight of *L. vannamei* significantly declined after fasting, but then increased immediately after re-feeding (Pascual et al., 2006; Sanchez-Paz et al., 2007). In the present study, histological damage of the hepatopancreas got remission after temperature return, confirming the shrimp’s self-repair ability.

### Plasma Metabolite Concentrations Change During Temperature Fluctuation

It has been widely accepted that protein acts as the main energy source for shrimp (New, 1976; Zhang et al., 2006; Cuzon et al., 2010). Research has shown that lipids are the main energy source of tilapia (*Oreochromis niloticus*) during long-time hypoxia stress (Li et al., 2018). In this study, the results showed that lipids (TC, the major components of lipids, supply and store energy) and protein (TP provides energy and transports various metabolites) in plasma responded more rapidly to temperature fluctuation, while Glu remained stable before 13°C and recovered to AT levels after temperature rose back to 28°C. It has been reported that the hepatopancreas is typically high in lipids and appears to be the main site for gluconeogenesis in decapod crustaceans (Hervant et al., 1999; Vinagre and Silva, 2011; Reyes-Ramos et al., 2018; Berry et al., 2019). Thus, combined with hepatopancreas histology and plasma results, we deduced that the increase of B-cells facilitates the gluconeogenesis to synthesize glucose from protein and lipid, by which shrimps maintain glucose demand under cold stress. However, after temperature dropped to 13°C, the rupture of hepatopancreas tubules causes lipids and proteins to enter hemolymph, resulting in an increase of lipid and protein content in plasma. The glucose content decreased at the same time due to the damage of the hepatopancreas.

It is known to all that nonspecific immunity plays an important role in the immune defense of aquatic animals. *L. vannamei* depends entirely on cellular and humoral immunity to prevent external injury (Iwanaga and Lee, 2005). ALP is directly involved in the transfer and metabolism of phosphoric acid groups in organisms and plays a significant role in the immune system against pathogens. The present study showed that ALP played a major role during the cold stress response in *Sparus aurata* and *L. vannamei*, and this is probably because ALP can help protect the hepatopancreas and hemolymph from cold-stress damage (Mateus et al., 2017; Peng et al., 2018). The activity of ALT in plasma can reflect the damage of the hepatopancreas (Jiang et al., 2014; Yan et al., 2016). It has been shown that various forms of stress can cause an increase in plasma ALT activity in fish (Cho et al., 1994), and it is responsive to temperature change in fish (Costas et al., 2012). In the present study, the ALP activity decreased to the lowest level, and the ALT activity increased to the highest level at 13°C, indicating that the shrimp was damaged at this temperature. However, there was no obvious change in the next 24 h when the temperature was kept at 13°C, but it increased significantly after the temperature rose back to 28°C. Thus, we deduced that shrimp has the ability to adapt low-temperature stress to a certain extent, and these results were consistent with those found in the hepatopancreas histological analysis.

### Differential Gene Expression About Unfolded Protein Response Pathway and Apoptosis During Temperature Fluctuation

UPR is a feedback regulatory system, capable of controlling the elimination of misfolded proteins in the ER, thereby maintaining the homeostasis of the endoplasmic reticulum. Appropriate ERS can activate UPR to improve the ER function and protect cells. But if the imbalance exceeds its regulating ability, it will lead to apoptosis. In invertebrates, apoptosis is also an effector factor of immune response. Environmental stresses such as temperature stimulation, pH changes, and toxic substances can induce apoptosis. In this study, the relative mRNA expressions of all UPR pathway- and apoptosis-related genes in the hepatopancreas and hemocytes were significantly changed during the cooling and re-warming process, which indicated that the UPR pathway and apoptosis participated in this process.

Previous studies indicated that shrimp can adapt to the environmental changes by self-regulation to a certain degree. In these studies, it was observed that the glutamate-oxalacetate transaminase and glutamate-pyruvate transaminase activities increased after shrimp were exposed to Zn for 7 days but declined after exposure for 14 and 28 days (Jui-Pin et al., 2008). Additionally, the lipid peroxidation levels in shrimp had no significant changes between 10 and 15 days after Cd exposure (Chiodi Boudet et al., 2015). In our study, the expressions of genes (GRP78, ATF6, IRE1, XBP1, PERK, eIF2α, and ATF4) in the hepatopancreas reached their highest level at 13°C instead of 13°C for 24 h. The plasma metabolites concentration analysis also showed that ALT activity got its highest point at 13°C, and the activity of ALT in plasma is inversely proportional to the health of hepatopancreas. This finding is consistent with previous studies and confirms the self-repair ability of shrimp. In addition, all these related gene expressions reached their highest level in 13°C in hepatopancreas, while in hemocytes their peak appeared in 13°C for 24 h. Thus, we deduced that the shrimp response to temperature fluctuation in the hepatopancreas may be relatively rapid compared to that that in hemolymph.

## Conclusions

In this study, protein and lipid were observed to be the main energy source of *L. vannamei* during temperature fluctuation. All the three UPR pathways were involved in temperature fluctuation process, and their responses in the hepatopancreas were relatively rapid compared to that in hemolymph. All the results suggest that *L. vannamei* can adapt to a certain level of temperature fluctuation by self-regulation. However, the detailed adaptation mechanism in *L. vannamei* still needs further study.

## Data Availability

All datasets generated for this study are included in the manuscript and/or the supplementary files.

## Ethics Statement

This study was carried out in accordance with the recommendations of the guidelines for animal care and use for scientific research in China. The protocol was approved by the Ministry of Agriculture and Rural Affairs of the China.

## Author Contributions

ZW performed the study, analyzed the results, and drafted the manuscript. YQ and JL coordinated the study. MY, JZ and LF set the experimental design. All authors reviewed and approved the final manuscript.

### Conflict of Interest Statement

The authors declare that the research was conducted in the absence of any commercial or financial relationships that could be construed as a potential conflict of interest.

## References

[ref1] Almohanna S. Y. Nott J. A. (1989). Functional cytology of the hepatopancreas of *Penaeus semisulcatus* (Crustacea: Decapoda) during the moult cycle. Mar. Biol. 101, 535–544. 10.1007/BF00541656

[ref2] Berry S. E. Simon C. J. Foote A. R. Jerry D. R. Wade N. M. (2019). Evaluation of baseline haemolymph biochemistry, volume and total body energetics to determine an accurate condition index in the black tiger shrimp, *Penaeus monodon*. Comp. Biochem. Physiol. B: Biochem. Mol. Biol. 228, 1–9. 10.1016/j.cbpb.2018.10.003, PMID: 30366056

[ref3] Cao S. S. Kaufman R. J. (2012). Unfolded protein response. Curr. Biol. 22, R622–R626. 10.1016/j.cub.2012.07.004 22917505

[ref4] Chen Y. H. He J. G. (2019). Effects of environmental stress on shrimp innate immunity and white spot syndrome virus infection. Fish Shellfish Immunol. 84, 744–755. 10.1016/j.fsi.2018.10.069 30393174

[ref5] Chen Y. H. Zhao L. Pang L. R. Li X. Y. Weng S. P. He J. G. (2012). Identification and characterization of inositol-requiring enzyme-1 and X-box binding protein 1, two proteins involved in the unfolded protein response of *Litopenaeus vannamei*. Dev. Comp. Immunol. 38, 66–77. 10.1016/j.dci.2012.04.005, PMID: 22554476

[ref6] Chiodi Boudet L. N. Polizzi P. Romero M. B. Robles A. Marcovecchio J. E. Gerpe M. S. (2015). Histopathological and biochemical evidence of hepatopancreatic toxicity caused by cadmium in white shrimp, *Palaemonetes argentinus*. Ecotoxicol. Environ. Saf. 113, 231–240. 10.1016/j.ecoenv.2014.11.019 25521337

[ref7] Cho C. Y. Hynes J. D. Wood K. R. Yoshida H. K. (1994). Development of high-nutrient-dense, low-pollution diets and prediction of aquaculture wastes using biological approaches. Aquaculture 124, 293–305. 10.1016/0044-8486(94)90403-0

[ref8] Collins P. (2010). Environmental stress upon hepatopancreatic cells of freshwater prawns (Decapoda: Caridea) from the floodplain of Paraná River. Nat. Sci. 2, 748–759. 10.4236/ns.2010.27094

[ref9] Costa C. Z. F. Rosa S. E. A. D. Camargo M. M. D. (2011). The unfolded protein response: how protein folding became a restrictive aspect for innate immunity and B lymphocytes. Scand. J. Immunol. 73, 436–448. 10.1111/j.1365-3083.2010.02504.x, PMID: 21204902

[ref10] Costas B. Ruiz-Jarabo I. Vargas-Chacoff L. Arjona F. J. Mancera J. M. Dinis M. T. (2012). Different environmental temperatures affect amino acid metabolism in the eurytherm teleost Senegalese sole (*Solea senegalensis* Kaup, 1858) as indicated by changes in plasma metabolites. Amino Acids 43, 327–335. 10.1007/s00726-011-1082-0 21947601

[ref11] Cottin D. Shillito B. Chertemps T. Tanguy A. Léger N. Ravaux J. (2016). Identification of differentially expressed genes in the hydrothermal vent shrimp *Rimicaris exoculata* exposed to heat stress. Mar. Genomics 3, 71–78. 10.1016/j.margen.2010.05.002 21798199

[ref12] Cuzon G. Cahu C. Aldrin J. F. Messager J. L. Stephan G. Mevel M. (2010). Starvation effect on metabolism of *Penaeus japonicus*. J. World Aquacult. Soc. 11, 410–423. 10.1111/j.1749-7345.1980.tb00135.x

[ref13] Dejean L. M. Martinezcaballero S. Kinnally K. W. (2006). Is MAC the knife that cuts cytochrome c from mitochondria during apoptosis? Cell Death Differ. 13, 1387–1395. 10.1038/sj.cdd.4401949, PMID: 16676005

[ref14] Duan Y. Liu Q. Wang Y. Zhang J. Xiong D. (2018a). Impairment of the intestine barrier function in *Litopenaeus vannamei* exposed to ammonia and nitrite stress. Fish Shellfish Immunol. 78, 279–288. 10.1016/j.fsi.2018.04.050, PMID: 29709590

[ref15] Duan Y. Wang Y. Dong H. Li H. Liu Q. Zhang J. (2018b). Physiological and immune response in the gills of *Litopenaeus vannamei* exposed to acute sulfide stress. Fish Shellfish Immunol. 81, 161–167. 10.1016/j.fsi.2018.07.018, PMID: 30017929

[ref16] Fan L. Wang A. Miao Y. Liao S. Ye C. Lin Q. (2016). Comparative proteomic identification of the hepatopancreas response to cold stress in white shrimp, *Litopenaeus vannamei*. Aquaculture 454, 27–34. 10.1016/j.aquaculture.2015.10.016

[ref17] Fan L. Wang A. Wu Y. (2013). Comparative proteomic identification of the hemocyte response to cold stress in white shrimp, *Litopenaeus vannamei*. J. Proteome 80, 196–206. 10.1016/j.jprot.2012.12.017, PMID: 23396037

[ref18] Han S. Y. Wang B. J. Liu M. Wang M. Q. Jiang K. Y. Liu X. W. (2018a). Adaptation of the white shrimp *Litopenaeus vannamei* to gradual changes to a low-pH environment. Ecotoxicol. Environ. Saf. 149, 203–210. 10.1016/j.ecoenv.2017.11.052, PMID: 29175347

[ref19] Han S. Y. Wang M. Q. Liu M. Wang B. J. Jiang K. Y. Wang L. (2018b). Comparative sensitivity of the hepatopancreas and midgut in the white shrimp *Litopenaeus vannamei* to oxidative stress under cyclic serious/medium hypoxia. Aquaculture 490, 44–52. 10.1016/j.aquaculture.2018.02.021

[ref20] He P. Wei P. Zhang B. Zhao Y. Li Q. Chen X. (2018). Identification of microRNAs involved in cold adaptation of *Litopenaeus vannamei* by high-throughput sequencing. Gene 677, 24–31. 10.1016/j.gene.2018.07.042 30016670

[ref21] Hervant F. Mathieu J. Culver D. C. (1999). Comparative responses to severe hypoxia and subsequent recovery in closely related amphipod populations (*Gammarus minus*) from cave and surface habitats. Hydrobiologia 392, 197–204. 10.1023/A:1003511416509

[ref22] Huang W. Ren C. Li H. Huo D. Wang Y. Jiang X. (2017). Transcriptomic analyses on muscle tissues of *Litopenaeus vannamei* provide the first profile insight into the response to low temperature stress. PLoS One 12:e178604. 10.1371/journal.pone.0178604, PMID: 28575089 PMC5456072

[ref23] Iwanaga S. Lee B. L. (2005). Recent advances in the innate immunity of invertebrate animals. J. Biochem. Mol. Biol. 38, 128–150. 10.5483/BMBRep.2005.38.2.128 15826490

[ref24] Jiang Q. Dilixiati A. Zhang W. Li W. Wang Q. Zhao Y. (2014). Effect of nitrite exposure on metabolic response in the freshwater prawn *Macrobrachium nipponense*. Cent. Eur. J. Biol. 9, 86–91. 10.2478/s11535-013-0167-4

[ref25] Jui-Pin W. Hon-Cheng C. Da-Ji H. (2008). Histopathological and biochemical evidence of hepatopancreatic toxicity caused by cadmium and zinc in the white shrimp, *Litopenaeus vannamei*. Chemosphere 73, 1019–1026. 10.1016/j.chemosphere.2008.08.019, PMID: 18809198

[ref26] Li E. Chen L. Zeng C. Yu N. Xiong Z. Chen X. (2008). Comparison of digestive and antioxidant enzymes activities, haemolymph oxyhemocyanin contents and hepatopancreas histology of white shrimp, *Litopenaeus vannamei*, at various salinities. Aquaculture 274, 80–86. 10.1016/j.aquaculture.2007.11.001

[ref27] Li M. Wang X. Qi C. Li E. Du Z. Qin J. G. (2018). Metabolic response of Nile tilapia (*Oreochromis niloticus*) to acute and chronic hypoxia stress. Aquaculture 495, 187–195. 10.1016/j.aquaculture.2018.05.031

[ref28] Madeira D. Mendonça V. Dias M. Roma J. Costa P. M. Larguinho M. (2015). Physiological, cellular and biochemical thermal stress response of intertidal shrimps with different vertical distributions: *Palaemon elegans* and *Palaemon serratus*. Comp. Biochem. Physiol. A Mol. Integr. Physiol. 183, 107–115. 10.1016/j.cbpa.2014.12.039, PMID: 25582544

[ref29] Mateus A. P. Costa R. Gisbert E. Pis P. Andree K. B. Estévez A. (2017). Thermal imprinting modifies bone homeostasis in cold challenged sea bream (*Sparus aurata*). J. Exp. Biol. 220:156174. 10.1242/jeb.156174 28733328

[ref30] Mori K. (2009). Signalling pathways in the unfolded protein response: development from yeast to mammals. J. Biochem. 146, 743–750. 10.1093/jb/mvp166 19861400

[ref31] Morishima N. Nakanishi K. Takenouchi H. Shibata T. Yasuhiko Y. (2002). An endoplasmic reticulum stress-specific caspase cascade in apoptosis. J. Biol. Chem. 277, 34287–34294. 10.1074/jbc.M204973200 12097332

[ref32] Nakka V. P. Gusain A. Raghubir R. (2010). Endoplasmic reticulum stress plays critical role in brain damage after cerebral ischemia/reperfusion in rats. Neurotox. Res. 17, 189–202. 10.1007/s12640-009-9110-5, PMID: 19763736

[ref33] New M. B. (1976). A review of dietary studies with shrimp and prawns. Aquaculture 9, 101–144. 10.1016/0044-8486(76)90055-7

[ref34] Pascual C. Sánchez A. Zenteno E. Cuzon G. Gabriela G. Brito R. (2006). Biochemical, physiological, and immunological changes during starvation in juveniles of *Litopenaeus vannamei*. Aquaculture 251, 416–429. 10.1016/j.aquaculture.2005.06.001

[ref35] Peng J. He P. Wei P. Zhang B. Zhao Y. Li Q. (2018). Proteomic responses under cold stress reveal unique cold tolerance mechanisms in the Pacific white shrimp (*Litopenaeus vannamei*). Front. Physiol. 9:1399. 10.3389/fphys.2018.01399 30483139 PMC6243039

[ref36] Peng J. Wei P. Chen X. Zeng D. Chen X. (2015). Identification of cold responsive genes in Pacific white shrimp (*Litopenaeus vannamei*) by suppression subtractive hybridization. Gene 575, 667–674. 10.1016/j.gene.2015.09.045 26407639

[ref37] Reyes-Ramos C. A. Peregrino-Uriarte A. B. Keni C. R. Valenzuela-Soto E. M. Lilia L. C. Gloria Y. P. (2018). Phosphoenolpyruvate carboxykinase cytosolic and mitochondrial isoforms are expressed and active during hypoxia in the white shrimp *Litopenaeus vannamei*. Comp. Biochem. Physiol. B: Biochem. Mol. Biol. 226, 1–9. 10.1016/j.cbpb.2018.08.001, PMID: 30107223

[ref38] Sanchez-Paz A. Muhlia-Almazan A. Yepiz-Plascencia G. (2007). Effect of short-term starvation on hepatopancreas and plasma energy reserves of the Pacific white shrimp (*Litopenaeus vannamei*). J. Exp. Mar. Biol. Ecol. 340, 184–193. 10.1016/j.jembe.2006.09.006

[ref39] Tao Y. Qiang J. Hui W. Pao X. U. Xinyu M. A. Zhao W. (2016). Acute toxicity of low-pH stress and its effect on enzyme activity and histological structure of gill and hepatopancreas in *Procambarus clarkii*. J. Fish. China 23, 1279–1289. 10.3724/SP.J.1118.2016.16056,

[ref40] Tobiume K. Matsuzawa A. Takahashi T. Nishitoh H. Morita K. Takeda K. (2001). ASK1 is required for sustained activations of JNK/p38 MAP kinases and apoptosis. EMBO Rep. 2, 222–228. 10.1093/embo-reports/kve046, PMID: 11266364 PMC1083842

[ref41] Verri T. Mandal A. Zilli L. Bossa D. Mandal P. K. Ingrosso L. (2001). D-glucose transport in decapod crustacean hepatopancreas. Comp. Biochem. Physiol. A Mol. Integr. Physiol. 130, 585–606. 10.1016/S1095-6433(01)00434-2, PMID: 11913469

[ref42] Vinagre A. S. Silva R. S. M. D. (2011). Effects of fasting and refeeding on metabolic processes in the crab *Chasmagnathus granulata* (Dana, 1851). Can. J. Zool. 80, 1413–1421. 10.1139/z02-122

[ref43] Wang X. Li E. Chang X. Qin J. G. Wang S. Chen X. (2016). Growth, body composition, ammonia tolerance and hepatopancreas histology of white shrimp *Litopenaeus vannamei* fed diets containing different carbohydrate sources at low salinity. Aquac. Res. 47, 1932–1943. 10.1111/are.12650

[ref44] Wang P. H. Wan D. H. Chen Y. G. Weng S. P. Yu X. Q. He J. G. (2013). Characterization of four novel caspases from *Litopenaeus vannamei* (Lvcaspase2-5) and their role in WSSV infection through dsRNA-mediated gene silencing. PLoS One 8:e80418. 10.1371/journal.pone.0080418, PMID: 24376496 PMC3871164

[ref45] Xu Z. Regenstein J. M. Xie D. Lu W. Ren X. Yuan J. (2018). The oxidative stress and antioxidant responses of *Litopenaeus vannamei* to low temperature and air exposure. Fish Shellfish Immunol. 72, 564–571. 10.1016/j.fsi.2017.11.016 29133253

[ref46] Xu J. Ruan L. Shi H. (2014). eIF2α of *Litopenaeus vannamei* involved in shrimp immune response to WSSV infection. Fish Shellfish Immunol. 40, 609–615. 10.1016/j.fsi.2014.08.016, PMID: 25149588

[ref47] Yan J. Chen S. Chang Q. Wang Z. Bin L. U. Liu C. (2016). Effects of antarctic krill meal replacing fish meal on growth performance, serum and liver biochemical indices and serum non-specific immune indices of juvenile spotted halibut (*Verasper variegatus*). Chin. J. Anim. Nutr. 28, 3503–3510. 10.3969/j.issn.1006-267x.2016.11.017

[ref48] Yepiz-Plascencia G. Galván T. G. Vargas-Albores F. García-Bañuelos M. (2000). Synthesis of hemolymph high-density lipoprotein β-glucan binding protein by *Penaeus vannamei* Shrimp hepatopancreas. Mar. Biotechnol. 2, 485–492. 10.1007/s101260000030, PMID: 11246415

[ref49] Yuan Z. Chen M. Wang J. Li Z. Geng X. Sun J. (2018). Identification of *Litopenaeus vannamei* BiP as a novel cellular attachment protein for white spot syndrome virus by using a biotinylation based affinity chromatography method. Fish Shellfish Immunol. 79:S1717046922. 10.1016/j.fsi.2018.05.003, PMID: 29738871

[ref50] Yuan F. H. Chen Y. G. Zhang Z. Z. Yue H. T. Bi H. T. Yuan K. (2016). Down-regulation apoptosis signal-regulating kinase 1 gene reduced the *Litopenaeus vannamei* hemocyte apoptosis in WSSV infection. Fish Shellfish Immunol. 50, 109–116. 10.1016/j.fsi.2015.12.003, PMID: 26806164

[ref51] Yuan K. He H. H. Zhang C. Z. Li X. Y. Weng S. P. He J. G. (2017). *Litopenaeus vannamei* activating transcription factor 6 alpha gene involvement in ER-stress response and white spot symptom virus infection. Fish Shellfish Immunol. 70, 129–139. 10.1016/j.fsi.2017.09.013, PMID: 28882789

[ref52] Zhang W. Chen B. Niu C. Yuan L. Jia H. Storey K. B. (2019). Response of the Chinese soft-shelled turtle to acute heat stress: insights from the systematic antioxidant defense. Front. Physiol. 10:710. 10.3389/fphys.2019.00710 31244677 PMC6562627

[ref53] Zhang P. Zhang X. Li J. Huang G. (2006). Swimming ability and physiological response to swimming fatigue in whiteleg shrimp, *Litopenaeus vannamei*. Comp. Biochem. Physiol., Part A: Mol. Integr. Physiol. 145, 26–32. 10.1016/j.cbpa.2006.04.014, PMID: 16843024

